# Disconnected or distressed? Exploring mobile phone use and unfinished nursing care among primary and hospital nursing professionals

**DOI:** 10.3389/fpubh.2026.1857828

**Published:** 2026-06-22

**Authors:** Cristina Jiménez-Moreno, Verónica V. Márquez-Hernández, Alba García-Viola, Vanesa Gutiérrez-Puertas, Lorena Gutiérrez-Puertas

**Affiliations:** 1 Faculty of Health Sciences, Department of Nursing, Physiotherapy and Medicine, University of Almeria, Almería, Spain; 2 Distrito Sanitario Almería, Almería, Spain

**Keywords:** hospital, mobile device, nomophobia, nursing, primary care, unfinished nursing care

## Abstract

**Background:**

The advancement of digital technologies has integrated mobile phone use into both daily and professional life. However, excessive use has led to the emergence of nomophobia (an irrational fear of being without one’s device), which may act as a source of distraction in healthcare settings, potentially compromising patient safety and the quality of care.

**Objectives:**

The aim of this study was to explore mobile phone use and the unfinished nursing care among Primary Care and Hospital nursing professionals.

**Methods:**

A descriptive cross-sectional study was conducted among 161 nursing professionals in Almería, Spain. The Nomophobia Questionnaire (NMP-Q) and the Unfinished Nursing Care Survey (UNC) were used. Data were analyzed using descriptive statistics, nonparametric tests (Mann–Whitney U test and Spearman’s correlation), and a multiple linear regression model (*p* < 0.05).

**Results:**

Participants showed moderate levels of nomophobia (*M* = 65.8; SD = 22.1) and a moderate-to-high prevalence of unfinished nursing care (*M* = 109.4; SD = 28.1). Significantly higher levels of nomophobia were found among hospital-based professionals (*p* = 0.041) and those who expressed a desire to change units (*p* = 0.042). No significant differences were observed by sex, although women reported higher scores. Regression analysis confirmed that nomophobia is a significant predictor of unfinished nursing care (*β* = 0.211; *p* = 0.002), explaining 24.7% of the variance in the model.

**Conclusion:**

Nomophobia shows a moderate prevalence among nursing professionals and is associated with unfinished nursing care. Mobile phone use may function as a cognitive avoidance mechanism in response to work-related stress; however, inappropriate use increases distractions and missed care. It is therefore necessary to develop institutional policies and awareness programs to regulate its use, in order to ensure safe, humanized, and efficient care.

## 1 Introduction

Advances in information technologies and the digitalization of communication media have facilitated access to information, generating a new style of communication and becoming an integral part of the population’s daily life (1–4). The use of mobile devices has accelerated the expansion of digital technology, enabling easy access to communication, entertainment, and information that appeals to all age groups (5, 6). However, excessive use of mobile phones is not without risks, as it is directly associated with changes in interpersonal communication styles, negative emotional responses, uncontrolled use, and the development of dependence (1, 3, 7). The term nomophobia, an abbreviation of “No-Mobile Phone Phobia,” refers to a situational phobia characterized by the fear of being unable to use a mobile phone or access its services, leading to anxiety when disconnected, as well as stress, sleep disturbances, and physical, social, and mental health problems (2, 3, 7, 8). It has been shown that constant attention to the mobile phone impairs concentration at work, leads to time loss, reduced motivation, and even occupational accidents (7, 9).

In the healthcare setting, mobile phone use facilitates professionals’ needs both for clinical purposes and for healthcare practice; however, it has also been shown to be used for spending time on social media (5, 10, 11). This situation negatively affects performance, efficiency, and the quality of care provided, as well as creating a gap between patient safety and nursing professionals’ risk assessment and safety behaviors (7, 12). In addition, it poses a risk of dehumanization and depersonalization of healthcare delivery (5).

Excessive mobile phone use in healthcare practice may negatively affect face-to-face communication, interdisciplinary collaboration, and the quality of patient care (13). In this regard, nursing professionals with high levels of nomophobia are at greater risk of making workplace errors, as this condition may interfere with appropriate perception and execution of clinical decision-making, potentially compromising patient safety (14).

**Figure 1:**
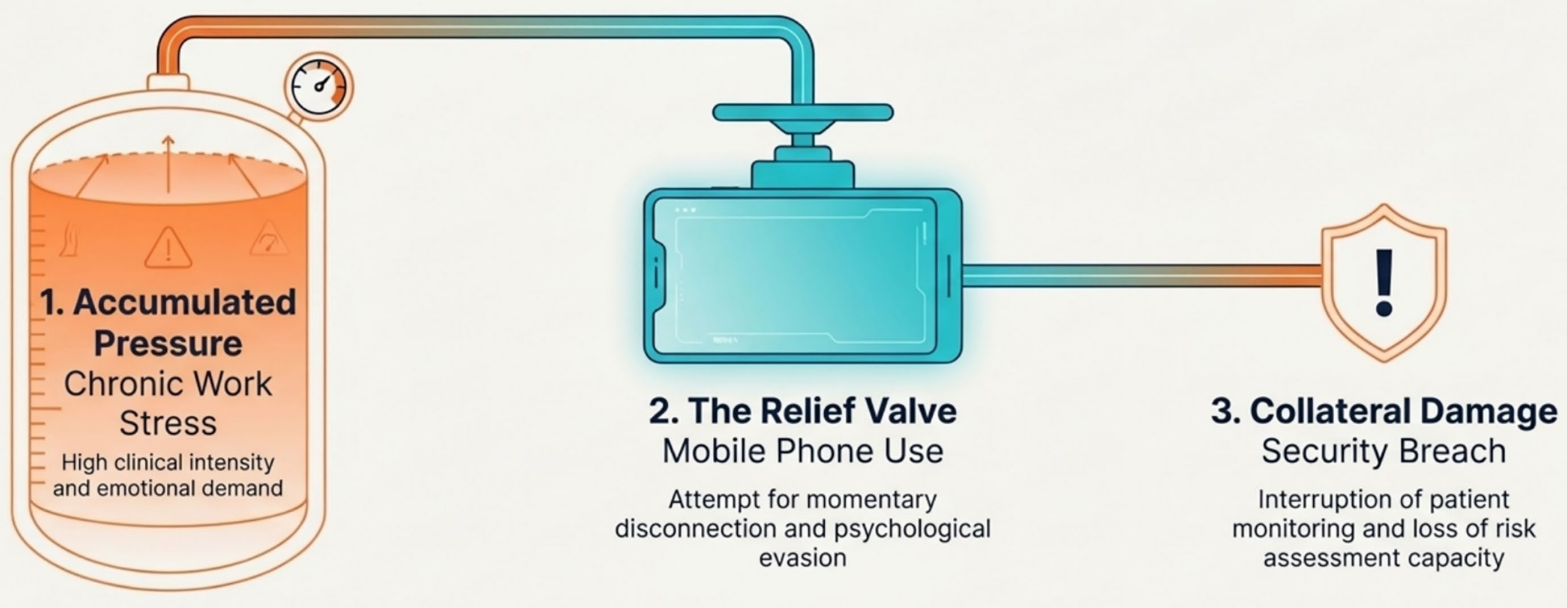
The mobile phone as an escape valve.

However, several studies have shown that mobile phone use may contribute to a reduction in perceived stress, generating a short-term sense of relief or satisfaction (5, 15). Mobile phone use during work breaks serves a recreational function that satisfies personal needs and provides relief during repetitive and routine work tasks (5). Likewise, the growing interest in mental health in the workplace has driven the development of digital applications aimed at managing stress and anxiety, reflecting an increasingly proactive approach to the psychological well-being of professionals (16). In this context, mobile phone use during scheduled breaks—such as between procedures or during lunch breaks—could represent a useful strategy for reducing work-related stress and, consequently, enhancing the performance of nursing professionals (5) (Figure 1).

In the hospital context, a high proportion of nursing professionals has been observed to use mobile phones in intensive care settings both for clinical improvement purposes and for personal use (10). Similarly, in Primary Care, a significant proportion of nursing professionals has been reported to use their mobile phones during working hours, with the main function being access to information on medications, procedures, and diseases (5). The clinical practice of healthcare professionals may be affected by levels of nomophobia, significantly compromising care continuity and quality, as it increases distractions and, consequently, the likelihood of errors (17).

Healthcare leaders have expressed concern regarding privacy breaches, distraction risks, and inappropriate mobile phone use in the workplace (10). This situation has led several healthcare institutions to call for the implementation of restrictive policies on mobile phone use by healthcare professionals in patient care areas or during working hours, reflecting awareness of the associated risks and aiming to prevent negative effects on the quality of care provided (5, 10).

Nomophobia is a clear and growing issue due to the rapid expansion of digital society (18). Likewise, nursing professionals play a fundamental role in delivering high-quality patient care (19). There is a clear need for further research into the impact of nomophobia on healthcare professionals in relation to the quality of care, as it is becoming a public health priority (20). Therefore, the main objective of this study was to explore mobile phone use and the unfinished nursing care among Primary Care and Hospital nursing professionals.

## 2 Method

### 2.1 Design

A descriptive study was conducted to explore mobile phone use and the unfinished nursing care among Primary Care and Hospital nursing professionals. The STROBE (Strengthening the Reporting of Observational Studies in Epidemiology) checklist guidelines were followed to ensure the quality of the study (21).

### 2.2 Participants

A convenience sampling strategy with voluntary participation was used. The inclusion criteria were: (a) actively employed nursing professionals; (b) access to a mobile device in the workplace; and (c) holding a long-term employment contract. The exclusion criteria were: (a) a prior diagnosis of psychological disorders such as diagnosed depression or anxiety; and (b) being on reduced working hours or sick leave.

The sampling frame comprised the complete list of nursing professionals meeting the inclusion criteria at the participating Hospital and Health District. All eligible professionals were contacted through institutional email with a direct link to the online survey. Although participation was voluntary, all eligible individuals had equal probability of being contacted and included. Of the 850 eligible professionals invited to participate, 161 nursing professionals from a Hospital and a Health District in Almería (Spain) completed the questionnaire, corresponding to a response rate of 18.9%.

### 2.3 Measurements

For data collection, sociodemographic variables of nursing professionals were considered. The sociodemographic characteristics included were: sex, age, workplace, type of employment contract, preference for changing units within the next six months, type of shift, years of experience, and age at which mobile device use began.

The Nomophobia Questionnaire (NMP-Q), developed and validated by Yildirim and Correia, consists of 20 items (22). In this study, the Spanish-adapted and validated version by Gutiérrez-Puertas et al. was used (23). The questionnaire is structured into four domains: (a) not being able to access information, consisting of 4 items (items 1–4); (b) giving up convenience, consisting of 5 items (items 5–9); (c) not being able to communicate, consisting of 6 items (items 10–15); and (d) losing connectivity, consisting of 5 items (items 16–20). Items are rated on a 7-point Likert scale, where 1 corresponds to “strongly disagree” and 7 to “strongly agree.” The total score is obtained by summing all item scores, with a range from 20 to 140 points, where higher scores indicate greater severity of nomophobia. In the original study, the instrument showed excellent internal consistency, with a Cronbach’s alpha of 0.95 for the overall scale and values ranging from 0.81 to 0.94 across its dimensions. In the present study, the questionnaire demonstrated an internal consistency of 0.917.

Additionally, the Unfinished Nursing Care Survey (UNC), developed by Bassi et al. (24), was used. The scale consists of two parts: Part A, which assesses unfinished care items through 21 items; and Part B, which examines the reasons for unfinished care through 18 items. Items are rated on a 5-point Likert scale, where 1 corresponds to “never” and 5 to “always.” Higher scores indicate a greater amount of missed or delayed care. In the original study, the scale showed a Cronbach’s alpha of 0.806 for the dimension related to reasons for unfinished care. In the present study, the scale demonstrated an internal consistency of 0.889, indicating adequate reliability for the assessment of unfinished nursing care.

### 2.4 Data collection

The principal investigator contacted the managers of the Hospital and the Health District to inform them about the aim and characteristics of the study. Subsequently, potential nursing professionals were informed through an information sheet outlining a brief introduction, the study objective, the voluntary nature of participation, and the possibility of withdrawing from the study, as well as participation via institutional email. The research collaborators were responsible for data collection and for addressing any questions raised by participants. At all times, contact with the principal investigator and co-principal investigator was made available.

An email was sent to each nursing professional with a brief description of the study, its purpose, and a direct link to the questionnaire for completion. Data were collected using an online survey. The online questionnaire consisted of the following sections: (a) study information, including details of the principal investigators and collaborating researcher, and informed consent; (b) sociodemographic characteristics of nursing professionals; and (c) questionnaires on the study variables. This study was conducted between January and April 2025.

### 2.5 Ethical aspects

This study was conducted in accordance with good clinical practice standards and applicable international and national regulations for biomedical research, particularly the Declaration of Helsinki, Spanish Law 14/2007 of July 3 on Biomedical Research, Regulation (EU) 2017/745 of the European Parliament and of the Council of April 27, 2017, and Spanish Law 3/2018 of December 5 on Personal Data Protection and the Guarantee of Digital Rights.

Participants took part voluntarily and were duly informed about the study’s objectives, procedures, risks, and benefits, and provided informed consent prior to participation. Confidentiality of data, participant anonymity, and the right to withdraw from the study at any time without consequences were ensured. In addition, permission was obtained from the original authors for the use of the scales employed in the research. The study was approved by the Provincial Research Ethics Committee of Almería (138/2023).

### 2.6 Data analysis

Data analysis was performed using IBM SPSS Statistics version 30.0 and the R statistical software. First, a descriptive statistical analysis of sociodemographic variables was conducted. For quantitative variables, mean, standard deviation, and range (minimum and maximum values) were calculated according to their distribution. For categorical variables, frequencies and percentages were computed.

The Kolmogorov–Smirnov test was used to determine the distribution of the variables. For hypothesis testing regarding equality of means in the main quantitative variables, the non-parametric Mann–Whitney U test was used. Relationships between the study variables were examined using Spearman’s correlation coefficient. A multiple linear regression model was performed to assess the relationship between problematic mobile phone use and relevant independent variables. Age, sex, and years of professional experience were included as covariates based on previous evidence suggesting their potential influence on both nomophobia and nursing care outcomes. In all cases, results were considered statistically significant at *p* < 0.05.

## 3 Results

### 3.1 Sociodemographic and employment characteristics of the participants

The sample consisted of 161 nursing professionals, of whom 88.2% were women (*n* = 142) and 11.8% were men (*n* = 19). The mean age of participants was 43.1 years (SD = 11.3), with a mean of 19.3 years of professional experience. Regarding workplace, 120 participants (74.5%) worked in hospitals and 41 (25.5%) in primary care centers. Most participants had full-time contracts (*n* = 139; 86.3%), while the remainder had part-time contracts at 75% (*n* = 18; 11.2%), 50% (*n* = 2; 1.2%), or 33% (*n* = 2; 1.2%).

Regarding the desire to change units, 22 professionals (13.7%) expressed interest in doing so, whereas 139 (86.3%) did not. In relation to shift type, 125 participants (77.6%) had rotating shifts, 23 (14.3%) had fixed morning shifts, 8 (5.0%) had fixed afternoon shifts, and 5 (3.1%) had fixed night shifts. The mean age at which participants began using mobile phones was 22.0 years (SD = 8.1; range 8–46). These data are presented in detail in Table 1.

**Table 1:** Sociodemographic and employment characteristics of the participants.

Variable	Category	n	%
Sex	Male	19	11.8%
	Female	142	88.2%
Workplace	Hospital	120	74.5%
	Primary Care Center	41	25.5%
Type of contract	Full-time	139	86.3%
	75% workload	18	11.2%
	50% workload	2	1.2%
	33% workload	2	1.2%
Willignes to change unit	Yes	22	13.7%
	No	139	86.3%
Shift type	Fixed morning shift	23	14.3%
	Rotating shift	125	77.6%
	Fixed afternoon shift	8	5.0%
	Fixed night shift	5	3.1%

Variable	M*	SD**	Range
Age	43.1	11.3	23–65
Years of experience	19.3	11.8	1–44
Age at first mobile phone use	22	8.1	8–46

*M, mean; **SD, standard deviation.

### 3.2 Nomophobia

Regarding nomophobia, the sample showed moderate levels, with a mean score of 65.8 (SD = 22.1) on a possible range of 20 to 140. By domain, the mean values were: domain 1 (*M* = 12.6; SD = 5.5), domain 2 (*M* = 16.5; SD = 7.1), domain 3 (*M* = 23.5; SD = 9.7), and domain 4 (*M* = 13.2; SD = 5.8).

No statistically significant differences were found according to sex (*U* = 1,530; *p* = 0.342), with men showing a mean nomophobia score of 61.2 (SD = 23.1) compared to 66.4 (SD = 21.9) in women. Professionals who expressed a desire to change units showed significantly higher levels of nomophobia (*M* = 72.9; SD = 24.1) compared to those who did not wish to change (*M* = 64.6; SD = 21.6) (*U* = 1943; *p* = 0.042).

**Figure 2:**
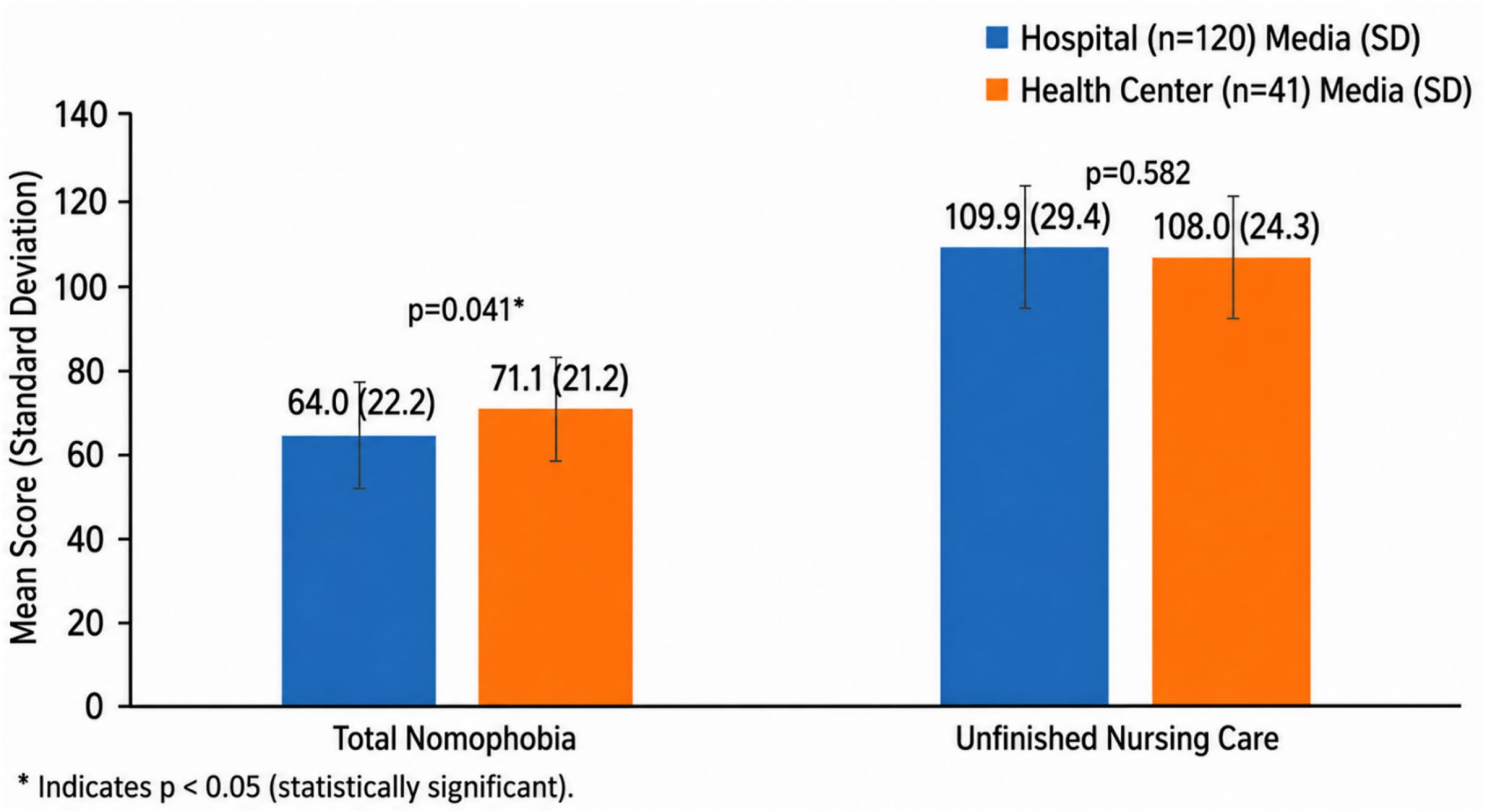
Comparative analysis by location: hospital vs. health center.

With regard to workplace, significant differences in nomophobia were identified according to work setting. Hospital-based professionals showed higher levels of nomophobia (*M* = 71.1; SD = 21.2) than those working in primary care centers (*M* = 64.0; SD = 22.2) (*U* = 1719; *p* = 0.041) (Figure 2).

### 3.3 Unfinished nursing care

The sample showed a moderate-to-high prevalence of unfinished nursing care, with a mean UNC score of 109.4 (SD = 28.1; range 39–177), with a mean of 62.5 (SD = 23.9) in the patient-related subscale (UNC A) and 46.9 (SD = 11.9) in the professional competence subscale (UNC B) (Table 2).

**Table 2:** Mean and standard deviation of nomophobia and unfinished nursing care, and their respective dimensions.

Variable	Media (DE)	Range
Total Nomophobia	65.8 (22.1)	20–140
Domain 1	12.6 (5.5)	4–28
Domain 2	16.5 (7.1)	5–35
Domain 3	23.5 (9.7)	6–42
Domain 4	13.2 (5.8)	5–35
Total UNC	109.4 (28.1)	39–177
UNC A (Patient relationship)	62.5 (23.9)	21–105
UNC B (Professional competence)	46.9 (11.9)	18–72

Considering sex, no statistically significant differences were found (*U* = 1,406; *p* = 0.762), with men obtaining a mean score of 107.8 (SD = 29.4) and women of 109.6 (SD = 28.0). Likewise, no statistically significant differences were found between the desire to change units and unfinished nursing care (*U* = 1874; *p* = 0.089), nor with regard to workplace (*U* = 2,220; *p* = 0.582) (Figure 2).

### 3.4 Nomophobia and unfinished nursing care

A moderate correlation was identified between nomophobia and unfinished nursing care (*ρ* = 0.310; *p* < 0.001), indicating that higher levels of nomophobia were associated with unfinished nursing care. Multiple linear regression analysis showed that nomophobia significantly predicted unfinished nursing care after controlling for age, years of experience, and sex. The model explained 24.7% of the variance [*F*(4,156) = 10.72; *p* < 0.001; adjusted R^2^ = 0.247]. Nomophobia had a effect on unfinished nursing care (*β* = 0.211; *p* = 0.002), whereas the other predictors were not statistically significant (Table 3).

**Table 3:** Multiple linear regression for predictors of unfinished nursing care.

Predictor	Standardized *β*	*p*-value
Nomophobia (TOTAL_NMPQ)	0.211	0.002*
Age	−0.085	0.292
Years of experience	0.127	0.087
Sex	0.052	0.446

* *p* < 0.05.

## 4 Discussion

The aim of this study was to explore the relationship between mobile phone use and the unfinished nursing care provided by primary care and hospital nursing professionals. In the current context, characterized by the rapid expansion of digital society and the increasing integration of mobile technologies into both personal and professional domains, profound changes have occurred in interpersonal communication patterns and information processing behaviors. These changes have contributed to the emergence and consolidation of phenomena such as nomophobia, understood as the fear or anxiety associated with being without access to a mobile phone or digital connectivity (6, 8, 25). This phenomenon has gained increasing relevance in recent years, particularly among populations with high levels of digital dependence, such as nursing professionals.

Within healthcare settings, the potential implications of excessive mobile phone use extend beyond individual behavior and may directly affect clinical performance and patient outcomes. Mobile devices, while offering significant advantages in terms of communication efficiency and access to clinical information, may also function as cognitive and attentional distractors during patient care activities. This dual nature raises important concerns regarding patient safety, as interruptions and divided attention during clinical tasks have been consistently associated with increased risk of errors, reduced situational awareness, and diminished quality of care (26). Simultaneously, healthcare professionals are routinely exposed to high levels of occupational stress, workload pressure, and emotional exhaustion. In this context, mobile phone use may serve not only as a communication tool but also as a maladaptive coping strategy, functioning as a mechanism of avoidance, emotional disengagement, or short-term relief from work-related demands (15, 16). Such patterns may reinforce problematic use behaviors over time, particularly in environments characterized by chronic stress.

In the present study, a moderate level of nomophobia was observed among nursing professionals. These findings suggest that, although extreme levels of dependency may not be predominant, a substantial proportion of professionals experience measurable anxiety related to mobile phone accessibility. Consistent with these results, Anggoro and Handiyani (27) reported that 63.7% of nursing professionals exhibited moderate levels of nomophobia, while 18.6% presented mild levels, highlighting a similar distribution pattern across different contexts. In contrast, other studies have indicated that healthcare professionals working in high-intensity or emotionally demanding environments may present a higher prevalence of severe nomophobia and stronger behavioral dependence on mobile devices (28). This variability across studies may reflect contextual differences in workload, organizational culture, and digital communication demands within healthcare systems.

Regarding sociodemographic variables, no statistically significant differences were found between nomophobia and sex, although women exhibited higher mean scores. This finding aligns with several previous studies that, despite not identifying significant sex-based differences, consistently report higher levels of nomophobia among women (27, 29). One possible explanation for this pattern relates to differences in usage behavior, as women are often reported to engage more frequently with social networking applications, which may foster greater emotional attachment and continuous connectivity behaviors. In contrast, men are more likely to use mobile devices for instrumental or task-oriented purposes, which may reduce emotional dependency. However, the literature remains inconsistent, as alternative evidence exists; for instance, Hamayatullah et al. (28) reported higher levels of mobile phone addiction among men, suggesting that cultural, occupational, and contextual factors may significantly moderate these associations.

A particularly relevant and novel finding of the present study was the statistically significant association between nomophobia and the desire to change work units. Although this relationship has not been directly explored in previous research, it may be theoretically interpreted through the framework of cognitive avoidance and stress-coping theories. In highly demanding or unsatisfactory work environments, individuals may engage in avoidant coping strategies aimed at reducing psychological discomfort. In this sense, mobile phones may function as an accessible and socially acceptable form of distraction or emotional escape, temporarily diverting attention from occupational stressors. Consequently, professionals who express a desire to change units may be experiencing higher levels of occupational dissatisfaction or emotional strain, which could be associated with increased reliance on mobile devices as a coping mechanism. This interpretation suggests a bidirectional relationship, where both workplace dissatisfaction and problematic mobile phone use may mutually reinforce one another. In addition, nursing professionals working in hospital settings exhibited higher levels of nomophobia compared to those in primary care. This finding may be explained by the specific organizational characteristics of hospital environments, which often involve higher patient acuity, faster decision-making processes, and greater reliance on rapid communication systems. Consistent with this interpretation, Al et al. (30) highlighted that the increased use of mobile applications as communication tools within hospital settings may contribute to higher levels of dependence and, consequently, greater nomophobic tendencies among healthcare professionals. Furthermore, the normalization of continuous connectivity in hospital workflows may inadvertently reinforce expectations of immediate responsiveness, further embedding mobile phone use into clinical routines.

With respect to nursing care quality, the present study identified a moderate-to-high prevalence of unfinished nursing care. This finding is particularly relevant, as unfinished care reflects a disruption in the continuity and completeness of essential nursing activities, which are fundamental to ensuring patient safety and optimal clinical outcomes. When critical tasks remain incomplete—especially those related to patient monitoring, education, and timely intervention—the risk of adverse events such as falls, hospital-acquired complications, and medication errors increases significantly. These deficiencies not only compromise patient safety but also reflect systemic pressures within healthcare environments, including staffing constraints, time limitations, and competing clinical priorities. The findings of the present study are consistent with those reported by Bruyneel et al. (31), who identified a similarly high incidence of unfinished nursing care among professionals working in intensive care units, where workload intensity and patient complexity are particularly elevated.

Moreover, the present study demonstrated that higher levels of nomophobia were associated with a greater prevalence of unfinished nursing care, suggesting a potential negative impact on the quality and continuity of patient care. This finding suggests that higher levels of mobile phone-related anxiety or dependence may be linked to poorer professional performance and reduced effectiveness in clinical care delivery. Consistent with this, Anggoro and Handiyani (27) reported that increased nomophobia is associated with diminished healthcare performance and, consequently, lower quality of care. Similarly, Cánovas-Zaldúa et al. (17) found that mobile phone-related interruptions during clinical practice are directly associated with reduced patient safety and poorer care outcomes. In addition, Al et al. (30) emphasized that the predominance of non-work-related mobile applications, such as social media platforms like Instagram, reflects a shift toward personal digital engagement during working hours. This behavior may contribute to attentional fragmentation, reducing cognitive focus on clinical tasks and ultimately compromising the quality and safety of patient care. Taken together, these findings support the hypothesis that excessive or maladaptive mobile phone use in healthcare settings may have meaningful implications for both professional performance and patient outcomes. However, the observed association should be interpreted with caution. Although problematic mobile phone use may contribute to attentional fragmentation and interruptions during clinical practice (17, 30), several occupational and organizational factors may independently confound this relationship. Workload intensity, staffing levels, burnout, occupational stress, and job dissatisfaction are well-established determinants of care quality that may simultaneously predispose professionals to maladaptive coping behaviors, including excessive mobile phone use, while also impairing care continuity and clinical performance (5, 15, 16, 31). Consequently, the observed association between nomophobia and unfinished nursing care may partially reflect the broader influence of an unfavorable occupational environment rather than an effect attributable solely to mobile phone dependence (27, 30).

The findings of this study should be interpreted in light of several limitations. First, the relatively small sample size of nursing professionals limits the statistical power of the analyses and reduces the generalizability of the findings to broader populations and different healthcare contexts. Second, the use of an online self-administered survey may introduce selection bias, as participation was voluntary and may have disproportionately attracted individuals with greater interest in digital health topics or mobile phone use behaviors. This may limit the representativeness of the sample and affect external validity. Additionally, the reliance on self-reported measures introduces the possibility of social desirability bias, in which participants may underreport undesirable behaviors, as well as recall bias, which may affect the accuracy of reported mobile phone usage and perceived impact on clinical practice. Furthermore, potentially relevant occupational variables such as workload, nurse-to-patient ratios, burnout, job satisfaction, and staffing levels were not assessed. These factors have been previously associated with unfinished nursing care and may have influenced the observed relationship between nomophobia and care outcomes. These methodological considerations should be taken into account when interpreting the results.

Future research should address these limitations by employing longitudinal study designs that allow for the examination of temporal and potentially causal relationships between mobile phone use and care quality. Larger and more diverse samples across different healthcare systems and professional groups would also enhance generalizability. Furthermore, the incorporation of mixed-methods approaches combining quantitative data with qualitative insights could provide a more comprehensive understanding of the behavioral, organizational, and psychological mechanisms underlying nomophobia in healthcare settings. Importantly, future studies should consider the use of objective measures of mobile phone usage, such as digital tracking applications or system logs, to reduce reliance on self-report data and improve measurement accuracy.

From an applied perspective, the findings of this study highlight the need for the development of institutional policies and guidelines regulating mobile phone use in clinical environments. Such strategies should aim to balance the legitimate clinical utility of mobile devices with the need to minimize distractions and maintain patient safety. In addition, training and awareness programs for nursing professionals may be beneficial in promoting responsible mobile phone use, enhancing self-regulation strategies, and increasing awareness of the potential impact of digital behaviors on clinical performance. Ultimately, these interventions could contribute to improving both the quality of care and patient safety, while supporting the well-being and professional functioning of healthcare workers in increasingly digitalized healthcare systems.

## 5 Conclusion

The evidence obtained indicates that nursing professionals present moderate levels of nomophobia, confirming that the irrational fear of being without access to a mobile device is a present and well-established phenomenon in the current clinical context. Likewise, a association was observed between nomophobia and unfinished nursing care. In this regard, mobile device use acts as a distractor and a source of interruptions, increasing patient safety risks and contributing to the occurrence of unfinished nursing care. Although no statistically significant differences were found according to sex, a tendency toward higher scores was observed in women. Additionally, the work environment emerged as a relevant factor, with higher levels of nomophobia detected among hospital-based professionals compared to those in primary care.

Overall, these findings highlight the need to develop and implement institutional strategies and training programs aimed at regulating mobile device use in clinical settings. Such interventions are essential to raise awareness among nursing professionals about the risks associated with distraction and to ensure safe, humanized, and high-quality patient care.

## Data Availability

The raw data supporting the conclusions of this article will be made available by the authors, without undue reservation.
